# Exposing the invader

**DOI:** 10.7554/eLife.79813

**Published:** 2022-05-30

**Authors:** Avraham N Bayer, Fred L Homa

**Affiliations:** 1 Divisions of Human Biology and Clinical Research, Fred Hutchinson Cancer Research Center Seattle United States; 2 Department of Microbiology and Molecular Genetics, University of Pittsburgh School of Medicine Pittsburgh United States

**Keywords:** MxB, herpes simplex virus, interferon, GTPase, capsid, defense proteins, Human

## Abstract

A restriction factor induced by interferons blocks the replication of herpesviruses by disassembling the capsid proteins surrounding their genome.

**Related research article** Serrero MC, Girault V, Weigang S, Greco TM, Ramos Nascimento A, Anderson F, Piras A, Hickford Martinez A, Hertzog J, Binz A, Pohlmann A, Prank U, Rehwinkel J, Bauerfeind R, Cristea IM, Pichlmair A, Kochs G, Sodeik B. 2022. The interferon-inducible GTPase MxB promotes capsid disassembly and genome release of herpesviruses. *eLife* **11**:e76804. doi: 10.7554/eLife.76804.

The immune system is a large network of organs, cells and molecules that work together to protect the body from disease-causing pathogens. It consists of an innate immune response (which activates macrophages and dendritic cells) and an adaptive immune response (which activates T cells and B cells).

Most cells in the body also have an intrinsic response against viruses, in which they express proteins that can detect the invading virus and block its replication. From the initial entry into a cell, through to the budding and release of new virus particles, intrinsic anti-viral factors can detect a broad range of viral molecules, including the RNA or DNA of the virus. In response, cells produce signaling proteins, such as interferons, to stimulate anti-viral defenses in neighboring cells and to target viral infections at various stages of their lifecycle.

For example, the restriction factor myxovirus resistance protein B (MxB) is activated by interferons, and has been shown to inhibit the replication of a broad range of viruses, including HIV and herpesviruses (Liu et al., 2013; Matreyek et al., 2014; Crameri et al., 2018; Schilling et al., 2018). However, its mechanism of action is poorly understood (Wilbourne and Zhang, 2021; Xie et al., 2021). Now, in eLife, Beate Sodeik and colleagues – including Manutea Serrero from Hanover Medical School as first author – report new insights into the interactions between MxB and herpesviruses (Serrero et al., 2022).

All herpesviruses consist of a capsid protein that surrounds their DNA, and a layer called the tegument that links the capsid with the membrane envelope engulfing the virus particle (Figure 1A; Dai and Zhou, 2018; Huet et al., 2016). Upon infection, the viral envelope fuses with the membrane of the host cell and releases the capsid and most of the tegument layer into the cell. These are then transported to the nucleus, where they can hijack the replication machinery of the host cell (Figure 1B).

**Figure 1.**
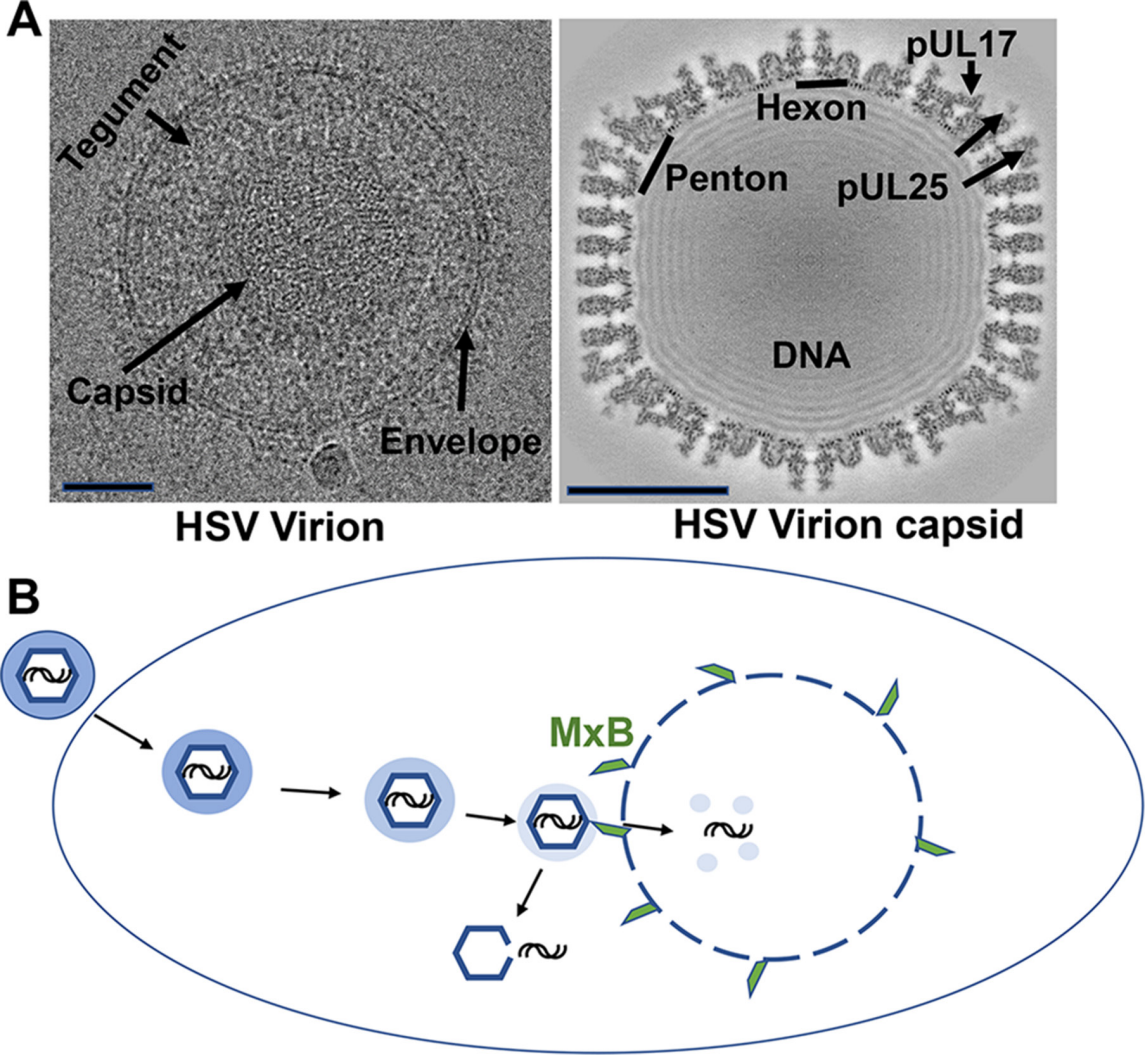
The structure and mechanisms of a herpes simplex virus. (**A**) Cryo-electron microscopy images of a herpes simplex virus (left) and a section through a three-dimensional reconstruction of the viral capsid (right). Herpesviruses consist of a protective capsid containing the viral DNA, and a tegument layer connecting the capsid and the membrane envelope surrounding the virus. Two subunits, hexon and penton, are located at the icosahedral vertices of the capsid, where three proteins (pUL17, pUL25, pUL36 – the latter is not visible on the map) form a complex that anchors the tegument layer to the capsid. Scale bars shown on lower left of each image represent 50 nanometers. (**B**) Schematic representation showing entry of the herpes virus into cells, following the fusion of the viral envelope with the host cell membrane. Once inside the cell, the capsid (blue hexagon) and its associated tegument proteins (shaded blue ring) traffic to the nucleus along microtubules, and dock at the pores of the cell nucleus to release the viral genome (black lines). The interferon-induced protein MxB (green) can only attach to the capsid when the tegument is absent (light blue shading) and divert the viral genome into the host’s cytoplasm by seemingly punching holes into the vertices of the capsid. The exposed viral genome may alert defense proteins to activate an anti-viral response.

To find out how anti-viral proteins inhibit the replication of herpes simplex virus, Serrero et al. used cell-free methods to reconstitute the complexes typically formed by these proteins and viral particles. To do so, they prepared protein extracts from ‘naïve’ macrophages and macrophages that had been exposed to an interferon, as these cells are known to mount a robust interferon response. These were then mixed with either capsids from extracellular viral particles containing varying amounts of tegument proteins, or tegument-free capsids from the nuclei of infected cells. The resulting complexes were characterized using quantitative mass spectrometry. This revealed MxB as one of the molecules interacting with the capsid, with a particular affinity for capsids lacking the tegument layer.

To test if MxB could also affect the stability of the capsid, the team (who are based at various institutes in Germany, the United States and the United Kingdom) bound the virus capsids onto electron microscopy grids and incubated them with cellular fluids extracted from naïve macrophages or macrophages that had been exposed to interferons. Electron microscopy revealed that capsids treated with the interferon-stimulated cell extracts were significantly more damaged and impaired compared to the capsids exposed to untreated cell extracts.

Moreover, further experiments using host cells from genetically modified lung tissue lacking MxB showed that extracts from these cells did not damage the capsid, highlighting its importance in capsid breakdown. In particular, MxB may attach to connector proteins (pUL17, pUL25, pUL36) that normally link the tegument and the capsid (Figure 1A). Furthermore, Serrero et al. found that when MxB was added to extracts from naïve cells that had not been activated by interferons, this caused the capsids to breakdown.

Perhaps the most intriguing result is that MxB works most efficiently on capsids lacking the tegument layer. In fact, MxB was unable to bind to capsids and destroy them when tegument proteins remained associated with the capsid. This suggests that tegument proteins shield the capsids from immune factors such as MxB, potentially by blocking their interaction with the connector proteins pUL17, pUL25 and pUL36.

The study of Serrero et al. further suggests that there is a fine balance within cells where loss of tegument proteins determines when capsids can be targeted by anti-viral factors, such as MxB. The binding of MxB to the tegument-free capsid and its partial destruction may in turn affect how new virus particles target the host cell’s nuclei. This could potentially disrupt the assembly of future capsids formed during viral replication, as MxB might engage with virions as they mature in the nucleus. In addition, the release of viral genomes from the damaged capsids may enhance the activation of defense proteins sensing the foreign particle, thereby amplifying the intrinsic immune responses of the host cell.

Since these experiments were done using cell-free assays, it remains to be seen whether MxB induces the breakdown of viral capsids in infected cells. A better understanding of how the tegument can shield the capsids from MxB could pave the way for new treatments against herpesviruses and other viral invaders.
